# Ward outings: providing patients with meal support in the real world

**DOI:** 10.1186/2050-2974-3-S1-P15

**Published:** 2015-11-23

**Authors:** Heather Litchfield

**Affiliations:** 1 Eating Disorders Unit - The Royal Melbourne Hospital, Melbourne, Australia

## 

Ward outings provide patients the opportunity to practice what they have learnt during their inpatient admission in a real-life context, to learn to feel comfortable eating out in public. The ward outing is for patients that are progressing well and have demonstrated that they can manage their meal plans on the unit and on leave.

The outing provides patients with an opportunity to challenge their eating disorder thoughts and beliefs around certain foods, by providing patients flexible options, whether the challenge is eating a sandwich in public, tackling a fear food or, having a meal that has not been eaten since the onset of a patient's illness.

During the outing the social worker provides patients with a positive social experience over a meal and activity of the patient's choosing. They conduct themselves as a positive role model; demonstrating to patients how to manage eating their meal in public, providing patients with a high level of support, encouraging the patients to order an appropriate meal, or, to consider challenging their eating disorder cognitions around certain foods. This is with the aim that patients will become more flexible with meal choices and be more willing to challenge their eating disorders.

